# Genetic evaluation of Mycobacterium bovis isolates with MIRU-VNTR and spoligotyping

**DOI:** 10.3906/sag-1910-138

**Published:** 2020-12-17

**Authors:** Nevin TUZCU, Fatih KÖKSAL

**Affiliations:** 1 Department of Biochemistry, Faculty of Pharmacy, Selçuk University, Konya Turkey; 2 Department of Medical Microbiology, Faculty of Medicine, Çukurova University, Adana Turkey

**Keywords:** *Mycobacterium bovis*, variable-number tandem repeats of mycobacterial interspersed repetitive units, spoligotyping

## Abstract

**Background/aim:**

Determining the epidemiological characteristics of *M. bovis* strains isolated from human and animal tuberculosis cases will assist in taking more appropriate and effective control measures in controlling tuberculosis originating from animals.

**Materials and methods:**

In this study, 32 *M. bovis* isolates of animal origin and 10 of human origin were isolated and identified in the Çukurova region between March 2011 and June 2012. The 12-locus MIRU-VNTR and spoligotyping methods were used.

**Results:**

Six different patterns were determined by spoligotyping and 10 by MIRU-VNTR. When both methods were used together, the number of patterns was found to be 28; MIRU4, MIRU26, MIRU31, and MIRU40 had the highest locus discrimination powers by MIRU-VNTR. The isolates concentrated in the SB0120 pattern at the rate of 42.85% in spoligotyping. By the same method, it was seen that 7 isolates were *M bovis ssp. caprae* pattern and 2 human isolates were *M. bovis BCG* pattern. Nevertheless, spoligotyping and MIRU-VNTR patterns showed that 5 *M. bovis* isolates of human origin were 100% compatible with isolates originating from cattle.

**Conclusion:**

In this study, we determined that the use of spoligotyping and MIRU-VNTR methods together was found to be more sensitive in the epidemiological analysis of *M. bovis* isolates.

## 1. Introduction

Tuberculosis caused by *Mycobacterium tuberculosis complex* (MTC) is an acute or chronic infection with different clinical manifestations. Species in MTC that causes tuberculosis are *M. tuberculosis* , *M. bovis* , *M. canettii* , *M. microti* , *M. africanum* , *M. caprae* , *M. pinnipedii* , *M. orygis* , *M. mungi* , dassie bacillus, bacille Calmette-Guérin (BCG) [1,2,3,4].

Cattle tuberculosis agent *M. bovis* is the mycobacteria with the widest host range, causing disease in animals and humans. *M. bovis* BCG was obtained by passaging *M. bovis* for 13 years and 230 times; it is generally used as a vaccine strain throughout the world [5,6].

The most important transmission source of *M. bovis* for humans is raw milk consumption, but infections also occur when humans come into direct contact with infected animals or inhale infectious aerosols. Infection from human to human is very rare, but it has been reported that such infection occurs, especially among immunocompromised humans [7,8]. *M. bovis* infections are very common in countries where the socioeconomic level is low and unpasteurized milk is widely consumed [9,10].

It is reported that tuberculosis cases caused by zoonotic strains like *M. bovis* have increased around the world in recent years, including Turkey. This fact increases the significance of determining and implementing methods that will ensure diagnosis of tuberculosis from animal-origin strains and will increase efforts for controlling the spread of animal-origin tuberculosis [11–13].

Clinical and pathologic symptoms of the infection caused by *M. bovis* are similar to those of infection caused by *M. tuberculosis* . This fact has affected the success rates of protection and treatment studies, as it has complicated the determination of the real incidence of *M. bovis* in patients with tuberculosis and has prevented the development of sufficient protection and treatment strategies for tuberculosis caused by this pathogen [14–16].

The spoligotyping method is a commonly used polymerase chain reaction (PCR) based on reverse dot blot hybridization method, and it is fast, simple, and repeatable [15]. The MTC genome contains a series of well-conserved 36 base pair direct repeats (DR) locus and nonrepetitive spacer sequences (34–41 base pairs) between the DR loci. The genetic relation among strains is determined by the absence or presence of the DR number of copies and spacer sequencing [17].

The mycobacterial interspersed repeat unit-variable number of tandem repeats (MIRU-VNTR) method is used in determining the repeat number and size of amplicons obtained through PCR performed by using primers that recognize regions that include the MIRU loci, the target sequences for PCR. The method has advantages because of its high specificity and repeatability among laboratories [17,18].

In this study, spoligotyping and 12-locus MIRU-VNTR methods were used to identify different strains of *M. bovis* isolated from human and cattle to better analyze the genetic diversity and determine the dominant types.

## 2. Material and methods

In order to determine the epidemiological features of *M. bovis* in the Çukurova region, 32 *M. bovis* isolates were identified with conventional methods; these processes were carried out on tissue and organs of 95 cattle which were determined after slaughter to have granulomatous pneumonia. The samples are transported to the laboratory under sterile conditions for bacteriologic culture. The sampled cattle were identified through postmortem evaluation out of 5018 cattle slaughtered for meat production between March 2011 and June 2012 in a local abattoir. During the same time period, clinical samples of patients prediagnosed with lung tuberculosis were submitted to the Çukurova University Region Tuberculosis Laboratory (THAUM) for tuberculosis diagnosis. Ten isolates were identified as *M. bovis* and *M. bovis BCG* from the sputum samples of these patients and taken from the Mersin University School of Medicine. Confirmed samples were further analyzed by using 12-locus MIRU-VNTR and spoligotyping methods.

### 2.1. Culture

Tissue samples were taken from the lesions of lung and lymph nodes of the cattle slaughtered in the abattoir, transported to the laboratory under sterile conditions, and decontaminated according to the protocol reported by Petroff [19]. Sputum samples taken from patients were prepared for culturing after homogenization and decontamination with the NALC–NaOH method [20].

Each sample was cultured in Lowenstein Jensen (LJ) medium enriched with 4 g of sodium pyruvate per liter (4 g/L) on 2 separate petri dishes, and samples were incubated at 37 °C [17–20]. At the end of the incubation process, the colonies were passaged on Middlebrook 7H9 broth medium. Erlich Ziehl Neelsen (EZN) dyed preparations were made with the colonies. Biochemical tests were applied when they were positive for acid-fast bacilli (AFB) [21].

It was determined by colony morphology that the growth of *M. bovis* was dysgonic on LJ agar based on the results from biochemical tests such as the niacin accumulation test (–), nitrate reduction reaction (–), and no color changes in bromcresol medium [21].

### 2.2. Spoligotyping

Spoligotyping was carried out according to the manufacturer’s instructions (Spoligotyping Kit; Isogen LifeScience, Utrecht, The Netherlands) and described by Kamerbeek et al. after DNA isolation from 42 *M. bovis* samples [22–24]. In order to obtain DNA from spoligotyping and MIRU-VNTR applied to all 42 *M. bovis* isolates, extraction was made from 7H9 broth medium and LJ medium by using a Mickle tissue disintegrator. DNA was stored at –20 °C until use.

**DRa:** 5’ - GGT TTT GGG TCT GAC GAC - 3’ (biotin labelled at the 5’ end);

**DRb:** 5’ - CCG AGA GGG GAC GGA AAC - 3’.

DRa and DRb primer pairs targeting the DR area were synthesized. The DRa primer was labelled with biotin and kept at +4 °C. The DRb primer was aliquoted into small amounts and kept at –20 °C. In each process, positive ( *M. bovis* , *M. bovis BCG* , *M. tuberculosis H37Rv* , or a clinical isolate whose genotype was known) and negative controls (dH2O) were used. PCR master mix (25 µL) consisted of 8.5 µL dH2O, 1.0 µL DMSO, 12.5 µL 2 × PCR master mix (Fermentas, Waltham, MA, USA), 0.25 µL DRa (25 pmol/µL), 0.25 µL DRb (25 pmol/µL), and 2.5 µL template DNA. Tubes containing PCR reaction mixture were placed in a Thermal Cycler device (Applied Biosystems, Beverly, MA, USA) and heat cycles were as follows: 5 min predenaturation at 95 °C, 40 cycles of 1 min denaturation at 94 °C, 1 min annealing at 55 °C, 45 s extension at 72 °C, and then 10 min final extension at 72 °C. Necessary buffer solutions were prepared for the denaturation step. Twenty µL PCR product was added to 150 µL 2 × SSPE/0.1% SDS. PCR products were denatured at 99 °C for 10 min. Membrane (Isogen) and sponge pad (Immunetics Plastic Cushion PC200, Immunetics Inc., Boston, MA, USA) were placed in a miniblotter (Miniblotter-3024). These products were then hybridized (FinePCR combi-SV120, FinePCR, Gunpo-si, South Korea) at 60 °C for 60 min after PCR products were added into the slots. Hybridized DNA was detected by chemoluminescence (Quantum-ST4 3020-WL/Blue/20M) at 450 nm. Hybrid regions were seen as black squares. By using the below octal coding key, results were converted to octal code consisting of 15 characters between 0 and 7. By using databases, groups and clades were determined for the obtained data.(Website http://www.pasteurguadeloupe.fr:8081/SITVITDemo/outilsConsultation.jsp [accessed 10 2012], http://www.miru-vntrplus.org [accessed 10 2012],, http://www.mbovis.org [accessed 10 2012] ).

□□□ = 0 □□■ = 1 □■□ = 2 □■■ = 3

■□ = 4 ■□■ = 5 ■■□ = 6 ■■■ = 7

■ = 1 □ = 0 Spacer 43

### 2.3. MIRU-VNTR

In order to determine the VNTR number of 12-locus MIRU for each strain, 12 PCR reactions were carried out [25,26]. For PCR, the reaction mixture was prepared as 2.170 μL dH2O, 0.250 μL DMSO, 3.125 μL 2× PCR master mix, 0.040 μL forward primer, 0.040 μL reverse primer, 0.625 μL DNA (2× PCR master mix: MgCl2 4 mM, dNTP mix (each dNTP 0.4 mM) 1.6 mM, Taq DNA polymerase 0.05 u/ μL). The thermal cycling protocol was as follows: 5 min predenaturation at 94 °C, 40 cycles of 30 s denaturation at 95 °C, 60 s annealing at 62 °C, 90 s extension in 72 °C, and then 10 min final extension at 72 °C.

The amplified DNA was visualized with a screening system (QUANTUM-ST4 3020–WL/BLUE/20M) after electrophoresis at 120 V for 0.6 h on a 2% agarose gel (SIGMA) stained with ethidium bromide. In order to determine band size, a 50–1000 bp marker (Fermentas) was used. Depending on the band size, at the end of 12-locus MIRU-VNTR typing, the allele repeat number for each MIRU locus was determined.

## 3. Results

In this study, in order to determine the epidemiologic features of *M. bovis* in the Çukurova region, 42 *M. bovis* isolates were evaluated with 12-locus MIRU-VNTR and spoligotyping methods. After these analyses, it was determined that both cattle and human isolates were grouped under 4 profiles. The most common profile was SB0120; interestingly, there were *M. bovis ssp. caprae* strains among cattle isolates. In clonal level genotyping performed with the 12-locus MIRU-VNTR method, it was determined that in all of the isolates there was no difference in the repeat numbers of MIRU2 (2), MIRU10 (2), MIRU16 (3), MIRU20 (2), MIRU23 (4), MIRU24 (2), MIRU27 (4), and MIRU39 (2) loci, but there were differences between repeat numbers of MIRU4, MIRU26, MIRU31, and MIRU40 loci. When these differences were taken into consideration, it was determined that a 100% relevant 10 MIRU-VNTR profile was obtained (as shown in Table 1).

**Table 1:** Spoligotyping patterns and 12 locus MIRU-VNTR profiles of 42 M. bovis isolates.

No ofisolates	Spoligotyping	Spoligotype(oktal)	Family	Spoligotype name		12 LokusMIRU-VNTR
				SpoIDB4	M.bovis.org	
H-1	■■□■■■■■□■■■■■■□■■■■■■■■■■■■■■■■■■	676773777777600	M.bovis BCG	SIT482	SB0120	232324244222
H-2	■■□■■■■■□■■■■■■□■■■■■■■■■■■■■■■■■■	676773777777600	M.bovis BCG	SIT482	SB0120	232324254322
H-3	■■□■■■■■□■■■■■■□■■■■■■■■■■■■■■■■■■	676773777777600	M.bovis	SIT482	SB0120	232324254322
H-6	■■□■■■■■□■■■■■■□■■■■■■■■■■■■■■■■■■	676773777777600	M.bovis	SIT482	SB0120	232324254322
H-7	■■□■■■■■□■■■■■■□■■■■■■■■■■■■■■■■■■	676773777777600	M.bovis	SIT482	SB0120	232324254321
H-9	■■□■■■■■□■■■■■■□■■■■■■■■■■■■■■■■■■	676773777777600	M.bovis	SIT482	SB0120	232324254323
B-2	■■□■■■■■□■■■■■■□■■■■■■■■■■■■■■■■■■	676773777777600	M.bovis	SIT482	SB0120	222324254423
B-6	■■□■■■■■□■■■■■■□■■■■■■■■■■■■■■■■■■	676773777777600	M.bovis	SIT482	SB0120	222324254322
B-7	■■□■■■■■□■■■■■■□■■■■■■■■■■■■■■■■■■	676773777777600	M.bovis	SIT482	SB0120	232324254222
B-11	■■□■■■■■□■■■■■■□■■■■■■■■■■■■■■■■■■	676773777777600	M.bovis	SIT482	SB0120	232324254422
B-13	■■□■■■■■□■■■■■■□■■■■■■■■■■■■■■■■■■	676773777777600	M.bovis	SIT482	SB0120	232324254323
B-17	■■□■■■■■□■■■■■■□■■■■■■■■■■■■■■■■■■	676773777777600	M.bovis	SIT482	SB0120	232324254222
B-20	■■□■■■■■□■■■■■■□■■■■■■■■■■■■■■■■■■	676773777777600	M.bovis	SIT482	SB0120	232324254322
B-21	■■□■■■■■□■■■■■■□■■■■■■■■■■■■■■■■■■	676773777777600	M.bovis	SIT482	SB0120	232324244322
B-27	■■□■■■■■□■■■■■■□■■■■■■■■■■■■■■■■■■	676773777777600	M.bovis	SIT482	SB0120	232324254322
B-24	■■□■■■■■□■■■■■■□■■■■■■■■■■■■■■■■■■	676773777777600	M.bovis	SIT482	SB0120	232324254223
B-29	■■□■■■■■□■■■■■■□■■■■■■■■■■■■■■■■■■	676773777777600	M.bovis	SIT482	SB0120	232324244222
B-32	■■□■■■■■□■■■■■■□■■■■■■■■■■■■■■■■■■	676773777777600	M.bovis	SIT482	SB0120	232324254322
H-4	■■□■■■■□□□□□■■■□■■■■■■■■■■■■■■■■■■	674073777777600	M.bovis	SIT685	SB0288	232324254323
H-5	■■□■■□■□□□□□■■■□■■■■■■■■■■■■■■■■■■	664073777777600	M.bovis	SIT683	SB0140	232324254321
B-1	□■□□□□□□□□□□□□□□■■■■■■■■■■■□■■■■■■	200003777377600	M.bovis subsp.caprae	SIT647	SB0418	232324254321
B-3	□■□□□□□□□□□□□□□□■■■■■■■■■■■□■■■■■■	200003777377600	M.bovis subsp.caprae	SIT647	SB0418	232324244322
B-8	□■□□□□□□□□□□□□□□■■■■■■■■■■■□■■■■■■	200003777377600	M.bovis subsp.caprae	SIT647	SB0418	232324254422
B-12	□■□□□□□□□□□□□□□□■■■■■■■■■■■□■■■■■■	200003777377600	M.bovis subsp.caprae	SIT647	SB0418	232324244322
B-14	□■□□□□□□□□□□□□□□■■■■■■■■■■■□■■■■■■	200003777377600	M.bovis subsp.caprae	SIT647	SB0418	232324254222
B-25	□■□□□□□□□□□□□□□□■■■■■■■■■■■□■■■■■■	200003777377600	M.bovis subsp.caprae	SIT647	SB0418	232324244223
B-31	□■□□□□□□□□□□□□□□■■■■■■■■■■■□■■■■■■	200003777377600	M.bovis subsp.caprae	SIT647	SB0418	232324254223
H-8	■■□■■□■□□□□□■■■□■■■■■■■■■■■■■■■■■■	664073777777600	M.bovis	SIT683	SB0140	232324244423
H-10	■■□■■□■□□□□□■■■□■■■■■■■■■■■■■■■■■■	664073777777600	M.bovis	SIT683	SB0140	232324244222
B-9	■■□■■□■□□□□□■■■□■■■■■■■■■■■■■■■■■■	664073777777600	M.bovis	SIT683	SB0140	232324254323
B-10	■■□■■□■□□□□□■■■□■■■■■■■■■■■■■■■■■■	664073777777600	M.bovis	SIT683	SB0140	232324254422
B-15	■■□■■□■□□□□□■■■□■■■■■■■■■■■■■■■■■■	664073777777600	M.bovis	SIT683	SB0140	232324254322
B-16	■■□■■□■□□□□□■■■□■■■■■■■■■■■■■■■■■■	664073777777600	M.bovis	SIT683	SB0140	232324254422
B-18	■■□■■□■□□□□□■■■□■■■■■■■■■■■■■■■■■■	664073777777600	M.bovis	SIT683	SB0140	232324254323
B-23	■■□■■□■□□□□□■■■□■■■■■■■■■■■■■■■■■■	664073777777600	M.bovis	SIT683	SB0140	232324254222
B-28	■■□■■□■□□□□□■■■□■■■■■■■■■■■■■■■■■■	664073777777600	M.bovis	SIT683	SB0140	232324254322
B-30	■■□■■□■□□□□□■■■□■■■■■■■■■■■■■■■■■■	664073777777600	M.bovis	SIT683	SB0140	232324254222
B-4	■■□■■■■□□□□□■■■□■■■■■■■■■■■■■■■■■■	674073777777600	M.bovis	SIT685	SB0288	232324254323
B-5	■■□■■■■□□□□□■■■□■■■■■■■■■■■■■■■■■■	674073777777600	M.bovis	SIT685	SB0288	232324254323
B-22	■■□■■■■□□□□□■■■□■■■■■■■■■■■■■■■■■■	674073777777600	M.bovis	SIT685	SB0288	232324254222
B-19	□■□□■□□□□□□□□□□□■■■■■■■■■■■□■■■■■■	220003777377600	-	-	-	232324254322
B-26	■■□■■■■□□□□□■■■■■■■■■■■■■■■■■■■■■■	674077777777600	-	-	-	232324254223

When clonal group distribution of 42 *M. bovis* isolates was performed in the study with the spoligotyping method, it was observed that 40 strains were placed into 4 groups (95.2%); a total of 18 (42.85%) isolates, including 2 human *M. bovis BCG* isolates, belonged to the most common spoligotype pattern, SB0120, while the spoligotype pattern that had the fewest members was SB0288, which included 4 isolates (9.52%). On the other hand, after comparing 2 isolates (4.76%) with the strains in the database used in the research, it was determined that they were orphan strains, not belonging to any of the groups in the study (shown in Table 2).

**Table 2:** Spoligotyping families and incidence of 42 M. bovis isolates.

Spoligotyping Families	Isolates Number	Incidence (%)
SB0120/SIT482	18	42.85
SB0140/SIT683	11	26.19
SB0418/SIT647	7	16.66
SB0288/SIT685	4	9.52
Unique	2	4.76

When profiles obtained with the 12-locus MIRU-VNTR method were taken into consideration, it was determined that the discriminative power of MIRU4 was low (0.01 < h < 0.11), while MIRU26, MIRU31, and MIRU40 had high discriminative power (0.25 < h), and other loci did not have discriminative power.

After evaluation of isolates with spoligotyping and 12-locus MIRU-VNTR methods, in terms of clonal relation, we observed that isolates formed 28 100% related subgroups with numbers ranging from 1–6; among these groups, the 18th group was the largest gene group containing 6 members, the 4th group was the second largest with 3 members, and 17 isolates belonged to independent groups with only a single member. It was also seen that there were 6 human isolates that had the same pattern as strains from animals; thus, there was a direct relationship between these human and animal strains.

## 4. Discussion

Tuberculosis is one of the most significant causes of death around the world; the frequency of this disease is closely related to socioeconomic conditions. Despite all of the control precautions used, including vaccination and effective medicines, it is still a significant threat to human health. This may be explained by factors such as low-sensitivity diagnosis methods, longer times for laboratory results, late commencement for treatments, insufficient treatment and medicines, treatment inconsistencies, and the lack of appropriate laboratory facilities in different regions. It is suggested that in order to prevent this disease, cheaper, faster, and more sensitive diagnostic methods should be developed, as well methods that will give epidemiologic results at the molecular level and will follow the transmission of MTC basils in the population.

Determining the epidemiologic features of *M. bovis* strains is very important for the control of both animal and human tuberculosis. However, because of ignorance of tuberculosis control and eradication, failure of applied programs and human consumption of contaminated products, *M. bovis* infection has been transferred to humans and has become a significant health threat throughout the world [25]. Today, in countries where human tuberculosis is rarely seen, animal tuberculosis is either eradicated or highly controlled. When this situation is taken into consideration, it is evident that in order to decrease the ratio of death and economic loss because of tuberculosis, it is necessary to determine the epidemiologic relationships at the clonal level among human and animal clinical isolates of *M. bovis* to carry out effective eradication of animal tuberculosis.

In this study, 5018 cattle were analyzed after slaughter and *M. bovis* strains were isolated with bacteriologic methods from tissue samples of 32 cattle (0.63%) out of 95 animals which were determined to have granulomatous pneumonia lesions in lungs and lymph nodes. This ratio was lower than the ratios determined by Ortatatlı et al. in Konya and Beytut in Kars [27,28]. This result in our study can be related to the fact that only *M. bovis* isolates were taken into consideration in our study.

In South Korea, Jeon et al. made a molecular study on 59 *M. bovis* isolates from cattle with granulomatous lesions using 12-locus MIRU, 3 exact tandem repeat (ETR) (A, B, C), 7 QUB (11a, 11b, 18, 26, 1895, 3232, 3336), and 2 VNTR (0424, 1955) regions in the study; they stated that 10 of these regions [MIRU (26, 31), ETR (A, B), Queen’s University Belfast (QUB) (18, 26, 1895, 3232, 3336) and VNTR (0424)] showed genetic polymorphism and that they obtained 12 different VNTR profiles in these regions. It was determined that the highest discriminative power belonged to the QUB3336 locus (h: 0.64), QUB 26, and MIRU 31 also had high discriminative power (h: 0.35). Four of these loci (MIRU26, ETR B, QUB 1895, VNTR 0424) had low discriminative powers (h: 0.02–0.05) and 3 different patterns were determined when complete 12-locus MIRU regions were studied [29].

Hilty et al. performed a study evaluating the discriminative power of 12-locus MIRU, with 3 ETR regions and VNTR 3232; they determined that ETR-A, B, C, MIRU26, and MIRU27 were highly polymorphic (h > 0.25); MIRU4 and VNTR3232 loci had a moderate level of discriminative power (0.11 < h < 0.25), MIRU16, MIRU20, and MIRU31 had low discriminative power (0.01 < h < 0.11), and MIRU2, MIRU10, MIRU23, MIRU24, MIRU39, and MIRU40 had no polymorphism [30].

Zuma et al. practiced genotyping on 224 *M. bovis* isolates with spoligotyping; the isolates were obtained from cattle in countries of South America. They determined 41 different spoligotype patterns and stated that 202 of the isolates (90%) formed 19 different groups: there were 96 isolates in the largest group and the most common pattern in cattle was spoligotype 34. The researchers used 154 of the isolates they analyzed in the study for comparing spoligotyping and restriction fragment length polymorphism (RFLP) (with PGRS probes); they reported that they obtained 31 different patterns in spoligotyping, 42 different patterns in RFLP, and 88 different patterns when both methods were used together [31].

In a study evaluating *M. bovis* in human tuberculosis epidemiology in the Aegean region, Çavuşoğlu and Yılmaz determined that of the 13 *M. bovis* isolates identified by spoligotyping from 482 MTC, there were 9 isolates (63.6%) of ST685 (SB0288), 1 isolate (7.7%) of ST1118 (SB0989), 1 isolate (7.7%) of ST820 (SB0856), and 2 isolates not in the databases [32]. Avsever et al. performed genotyping on 6 *M. bovis* isolates with the spoligotyping method; the isolates were obtained from 4 cattle and 2 goats in the same region. They defined all isolates as SIT 685. The differences between the spoligotype pattern determined in our study and the abovementioned studies are thought to be due to regional differences (Çukurova and Aegean) and the number of *M. bovis* isolates [33].

In Oral and Köksal’s study in the Çukurova region, isolations were produced from sputum, bronchoalveolar lavage, and biopsy materials taken from patients with lung tuberculosis; they defined 467 isolates as MTC through spoligotyping and the 12-locus MIRU-VNTR method. After spoligotyping, they placed 443 isolates in 21 groups; the largest group comprised the T1 family and contained 239 members, of which 2 isolates had *M. bovis* (Bovis1) (0.4%) [22] .

Duarte et al. applied spoligotyping on 181 *M. bovis* and *M. bovis ssp. caprae* strains and determined 12 groups; they also applied the 8-locus MIRU-VNTR method on the same strains and determined 87 different profiles. On the other hand, researchers reported that VNTR3232, QUB11a, ETR-B, and ETR-A loci had the highest discriminative power (h: 0.96). Experience and substructure are also significant indicators in the use of methods. We did not use ETR regions in this study, but we know that the profile we obtained is an accepted combination used in determining the level of clonal relation [34].

When the clonal distribution of 42 *M. bovis* isolates was analyzed in this study, it was determined with the spoligotyping method that 40 strains (95.2%) were in 4 groups; spoligotype pattern SBO120 was the pattern in which all of the *M. bovis BCG* strains were grouped, totaling 42.85% (18 isolates). The second most common spoligotype pattern was SB0140, containing 11 isolates (26.19%); the third spoligotype pattern was SB0418, containing 7 isolates (16.66%); the fourth and last spoligotype pattern was SB0288, which contained 4 isolates (9.52%). In our study, the SB0120 spoligotype pattern was determined to be the most common spoligotype pattern; this result is similar to those of the other studies mentioned above. The other patterns in our study are also consistent with the results in the literature. On the other hand, after comparing 2 strains (4.76%) obtained in the study with strains in the literature, it was seen that they did not belong to any of the groups; they are orphan strains.

In conclusion, it is thought that spoligotyping is easier and more repeatable than the MIRU-VNTR method; thus, it can be beneficial for developing protection and control strategies and evaluating the success of applied strategies. When it is combined with the MIRU-VNTR method, it will be possible to make smaller-scaled projections that are epidemiologically more detailed. On the other hand, determining *M. bovis ssp. caprea* in strains obtained from these animals shows that this strain should also be taken into consideration while preparing tuberculosis control programs.
